# Ayurvedic formulations Guduchi and Madhuyashti triggers JNK signaling mediated immune response and adversely affects Huntington phenotype

**DOI:** 10.1186/s12906-022-03724-9

**Published:** 2022-10-12

**Authors:** Surabhi Singh, Madhu G. Tapadia

**Affiliations:** Cytogenetics Laboratory, Department of Zoology, Banaras Hindu University, Varanasi, Uttar Pradesh 221005 India

**Keywords:** Guduchi, Madhuyashti, Ayurveda, Immune response, Neurodegeneration, JNK signaling

## Abstract

**Background:**

Huntington’s disease manifests due to abnormal CAG trinucleotide expansion, in the first exon of the *Huntingtin* gene and disease progression involves genetic, immune, and environmental components. The pathogenesis is characterized by the formation of Inclusion Bodies, disruption of neuronal circuitry, cellular machinery, and apoptosis, resulting in gradual and progressive loss of neuronal cells, ultimately leading to nervous system dysfunction. Thus, the present study was conducted to assess the effect of two Ayurvedic formulations, Guduchi and Madhuyashti, on Huntington’s phenotype, using *Drosophila* as a model system.

**Method:**

The Huntington phenotype was ectopically induced in the *Drosophila* eye using the UAS-GAL4 binary system and the effect of the two Ayurvedic formulations were assessed by feeding the progenies on them. Degeneration was observed microscopically and Real Time-PCR was done to assay the alterations in the different transcripts of the innate immune pathways and JNK signaling pathway. Immunostaining was performed to assay different gene expression patterns.

**Result:**

The present study shows that Guduchi and Madhuyashti, endowed with immunomodulatory and intellect promoting properties, aggravates polyQ mediated neurodegeneration. We provide evidence that these formulations enhance JNK signaling by activating the MAP 3 K, dTAK1*,* which regulates the expression of *Drosophila* homologue for JNK. Sustained, rather than a transient expression of JNK leads to excessive production of Anti-Microbial Peptides without involving the canonical transcription factors of the Toll or IMD pathways, NF-κB. Enhanced JNK expression also increases caspase levels, with a concomitant reduction in cell proliferation, which may further contribute to increased degeneration.

**Conclusion:**

This is a report linking the functional relevance of Guduchi and Madhuyashti with molecular pathways, which can be important for understanding their use in therapeutic applications and holds promise for mechanistic insight into the mammalian counterpart.

**Supplementary Information:**

The online version contains supplementary material available at 10.1186/s12906-022-03724-9.

## Background

Age-related diseases, especially neurodegenerative disorders have emerged as major contributors to pathological problems, leaving behind the incidence of infectious diseases due to increasing awareness toward health and hygiene. One of the neurodegenerative disorders caused due to CAG trinucleotide repeat sequence expansion (PolyQ) includes Huntington’s Disease (HD) [30], where CAG inherited codon reiteration associated with dynamic mutations, increases the number of glutamine residues beyond a certain threshold, thus proving to be fatal. PolyQ diseases are characterized by the formation of Inclusion bodies (IBs) in the affected neurons, which sequester a variety of cellular or nuclear components, leading to pathogenicity, cellular dysfunctioning, axonal transport defects and apoptosis in the neuronal cells [13, 21, 34].

Erroneous triggering of immune signaling pathways has emerged as a crucial component of pathogenesis in many neurodegenerative diseases [6, 19], including HD [12]. Lower organisms such as *Drosophila*, rely only on innate immunity comprising of cellular response through phagocytosis, encapsulation or melanisation, and humoral response through the production of Anti-Microbial Peptides (AMP). IMD and Toll pathways participate in the production of AMPs by activating the NF-kB transcription factors, Relish and Dorsal/DIF respectively [7, 14, 15]. Jun N-terminal kinase (JNK) signaling has been linked to stress response, cell migration, apoptosis, and immune responses in both insects and mammals [36, 43]. JNK regulates AMP expression immediately upon infection and is independent of Relish [11, 28, 41]. Activation of the apoptotic pathway could be the ultimate consequence leading to the death of neurons. AMP transcription has also been observed to be regulated by the JAK-STAT pathway [5].

Conventional medicines are used for the holistic well-being of humans and the goodness of Ayurvedic formulations is closely coupled with influencing the physiological status of humans. The molecular mechanisms that link these processes are, however, not understood very clearly. Here we present results of two different Ayurvedic formulations, Guduchi and Madhuyashti, when used in a Huntington model in *Drosophila*. Guduchi or *Tinospora cordifolia* and Madhuyashti, also known as *Glycyrrhiza glabra,* are known to possess immunomodulatory properties, thus promoting disease free long life [9, 37]. Studies show that these two formulations have a neuroprotective role [3], and are involved in improving learning and memory in case of scopolamine-induced dementia, cognition deficits, treatment of vertigo, in improving behavior disorders, mental deficit, and intelligence quotient (IQ) levels [33, 40]. However, contrary to the expected results, in this paper we present evidence that under conditions of Huntington’s disease, these formulations, at the concentrations used, adversely affect the pathogenesis, by activating the TAK1 mediated JNK signaling, thus affecting AMP synthesis and caspase activation in *Drosophila* model.

## Methods

*Drosophila* was reared on standard agar-cornmeal-sugar-yeast food at 24 ± 1 °C. Guduchi and Madhuyashti powders were obtained from Arya Vaidya Sala (Kottakkal, Kerala, India) and were mixed with standard food separately at a concentration of 0.5% [37, 38], for rearing experimental larvae and/or flies. Controls were reared on un-supplemented regular food. In all experiments, first instar larvae were collected from flies maintained on regular food, and the same batch of food was used in regular and formulation supplemented medium to eliminate differences due to food, and likewise, all larvae/adults for a given experiment were derived from a common pool of eggs and reared in parallel on the regular or formulation supplemented food. In all experiments, eggs were collected from fly stocks that were always reared on regular food. All experiments were done in 3 independent triplicates and for each genotype, more than 300 flies were observed.

### Fly stocks

(Unless mentioned, all fly stocks are from Bloomington Stock Centre)

*Oregon R*^*+*^ Wild type*; UAS-CL1–GFP* [25] (Kind gift from Dr. Udai Bhan Pandey, USA); *GMR-GAL4:UAS-127Q/CyO-GFP:* [12]; *w1118; +;UAS-bsk*^*DN*^ [39]; w*;UAS-Tak1^*DN*^*/Cyo-GFP.*

The following stocks are referenced in *[*29*]* *UAS-Tak1RNAi/TM3,Ser*, *y,sc; Cecropin RNAi; +*, *y,v;+; Metchnikowin RNAi/TM3,Sb*, y*,v; +; Defensin RNAi*, *y,v; +; Diptericin RNAi*. Appropriate crosses were set up to obtain the required progenies.

### Photomicrographs of adult eyes

Flies of the desired genotypes were etherized and their eyes were photographed for external morphology, using a Sony Digital Camera (DSC-75) attached to a Zeiss Stemi SV6 stereobinocular microscope.

### Nail polish imprints

Eye imprints to create the exact replica of the external surface of the eye was done using nail polish and examined by DIC microscopy [1].

### RNA isolation and qPCR

Desired tissues of late third instar larvae were dissected out in Phosphate Buffered Saline (PBS). Total RNA was isolated using the Trizol as per the manufacturer’s (Ambience, India) instructions. RNA pellets were re-suspended in nuclease-free water and cDNA was synthesized; real time PCR (RT-qPCR) was carried out using appropriate primers and SYBR-Green dye on 7500 Real Time PCR System (Applied Biosystems) with the 7500 software v2.0.4. The primers used for G3PDH and AMPs are described earlier [37]:

(i). Relish: Forward 5′ CCCATGTCCCAAAGGCTGAT 3′
  Reverse 5′ GCTGGAGATCAAGTCCGAGG 3′
(ii). Imd: Forward 5′ TTTTCGACACAAGTATCACAACTT 3′
  Reverse 5′ GAGAAGAGACGAACACGGGA 3′
(iii). Fadd: Forward 5′ CATCTTTGTCGCTCTCCAGC 3′
  Reverse 5′ CTACCCGCACGACTTGAAGT 3′.
(iv). Tak1: Forward 5′ ACACGGACTGAATACGCTTAGA 3′
  Reverse 5′ CACAATGCGTAGTCTCGTGC 3′.
(v). Tab2: Forward 5′ GCAGCCGATTACAGTCCTCA 3′
  Reverse 5′ GCCACTATCGAGCGTCAGAA 3′.
(vi). Spatzle: Forward 5′ ACTAATTACTTGCATTTCTAGCTTAGG 3′
  Reverse 5′ AAGAAGTTTCACCCACCACAGAATG 3′.
(vii). Tube: Forward 5′ TTCTGCTCTCAGGACGCAAG 3′
  Reverse 5′ AAGCTTCAACGGCTACCACA 3′.
(viii). Myd88: Forward 5′ GAGGTACGTGTTCTCCGGAC 3′
  Reverse 5′ CGACATGCTAATGGGCGTTC 3′.
(ix). Pelle: Forward 5′ GAGGTACGTGTTCTCCGGAC 3′
  Reverse 5′ CGACATGCTAATGGGCGTTC 3′.
(x). Cactus: Forward 5′ TCGCTACTGTCGTAATCACTGT 3′
  Reverse 5′ ACCAGTTTGCGTGCATCATG 3′.
(xi). Dorsal: Forward 5′ GCCGCCTCAATGTCCTTCTT 3′
  Reverse 5′ CTGCAAGAAGGGCGTCTGTA 3′.
(xii). Hemipterous: Forward: 5′- GCATCAATCAATTCCCGCCA − 3′,
  Reverse: 5′- GCTCTGCCAGCACTGATGTA − 3′.
(xiii). Basket: Forward: 5′- GCATCAATCAATTCCCGCCA − 3′,
  Reverse: 5′- GCTCTGCCAGCACTGATGTA − 3′

### Calculation of ∆∆CT values

The ΔΔCT method is used for relative quantification for qPCR experiments. The threshold cycle (CT) is the cycle at which the fluorescence level reaches the threshold. This method directly uses the CT information generated from a qPCR system to calculate relative gene expression in target and reference samples, using a housekeeping gene as the normalizer. A target sample here is a sample from Guduchi or Madhuyashti fed larvae, while a reference sample is larvae fed on control food. G3PDH has been used as an internal control to normalize the PCRs because their expression levels remain relatively stable in response to any treatment.

$$\Delta \Delta \mathrm{CT}=\Delta \mathrm{CT}\;\left(\mathrm{a}\;\mathrm{target}\kern0.17em \mathrm{sample}\right)\hbox{-} \Delta \mathrm{CT}\;\left(\mathrm{a}\;\mathrm{reference}\kern0.17em \mathrm{sample}\right)$$

### Immunostaining of tissues

Desired tissues were dissected out in PBS and fixed in freshly prepared 4% paraformaldehyde for 20 mins at RT and processed further for immunostaining as described [31]. The different primary antibodies used were: (1) rabbit anti-HA (1:100) (Molecular probes), (2) rat anti-ELAV (Rat-Elav-7E8A10) (1:100) (DSHB), (3) mouse anti-mab22c10 (anti-Futsch) (1:100) (DSHB), (4) rabbit anti-p-JNK (SAPK) (9251) (1:100) (Cell Signaling Technology), (5) mouse anti-Relish (anti-Relish-C 21F3) (1:100) (DSHB), (6) rabbit anti-Caspase3 (C8487) (1:100) (Sigma), rabbit anti-Phosphohistone3 (05–1336) (1:1000) (Merk). Appropriate secondary antibodies conjugated either with Cy3 (1:200, Sigma-Aldrich) or Alexa Fluor 488 (1:200, Molecular Probes) were used to detect the primary antibody. The tissues were counterstained with DAPI (1 μg/ml; Sigma Aldrich, India), mounted in DABCO (Sigma), and examined under LSM510 Meta Zeiss laser scanning confocal microscope analyzed using LSM software and assembled using Adobe PS 7.0.

### Statistical analysis

For all the figures (Figs. 2, 3, 4, 6 and 8) which has quantification, it was done for the areas below morphogenetic furrow as GMR-GAL4 expresses only there. All the experiments were done in triplicates and for each experiment about 20–25 eye discs were scored. Sigma Plot 11.0 was used for statistical analyses. Comparisons between groups were made using Student’s t-test.

## Results

### Dietary supplement of Guduchi and Madhuyashti aggravates degeneration caused by expanded polyQ repeats

**Fig. 1:**
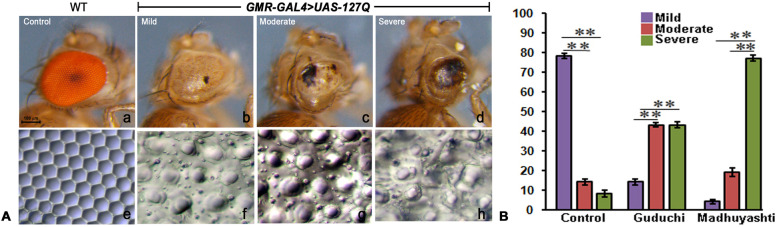
Guduchi and Madhuyashti feeding exaggerate polyQ mediated neurodegeneration in *GMR-GAL4 > UAS-127Q/CyO-GFP* flies. **A** Eyes classified into mild (**b**), moderate (**c**) and severe (**d**) types in comparison to normal wild type (**a**). Nail polish imprints of wild-type eyes (**e**) showing proper ommatidial organization, which was disrupted in mild (**f**), moderate (**g**) and severe (**h**) categories. **B** Histogram showing mean percentage (+SD) for the eye phenotype mild (purple), moderate (red) and severe (green) in *GMR-GAL4 > UAS-127Q/CyO-GFP* flies when grown on regular food, Guduchi and Madhuyashti. Y-axis represents percentage of eyes observed for each phenotype and X-axis represents different feeding regimes. Scale bar in **a** represents 100 μm and applies from **a-h**. ** represents *P* ≤ 0.001 using Student’s t-test

*Drosophila* compound eyes are made up of regular units known as ommatidia and the expression of polyQ repeats, specifically in the developing eyes, disrupts the regular arrays of ommatidia due to the polyQ-toxicity induced neurodegeneration. GMR-GAL4 was used to drive the expression of 127 polyQ repeats under UAS element (UAS-127Q) to induce degeneration which showed variable expression and the extent of variation was categorized (Fig. 1A) into mild (b), moderate (c), and severe (d), based on the extent of loss of pigmentation, the formation of necrotic patches, and collapsed eyes, in comparison to wild-type adult eyes which showed a normal array of the hexagonal lattice (a). The nail polish imprint technique further confirmed the categorization of the degeneration (e, f, g, h). To analyze the effect of Guduchi and Madhuyashti on neurodegeneration, first instar larvae of *GMR-GAL4 > UAS-127Q/CyO-GFP* were fed on the formulation or regular food and the consequences on degeneration on the adult eye phenotype was observed by counting the number of flies in each category (Fig. 1B). Among the flies fed on regular food, the highest percentage of flies was observed in the mild degeneration category and significantly less in moderate or severe. On the other hand, feeding on Guduchi supplemented food, significantly increased the flies in the moderate and severe categories to a similar extent, with a concomitant decrease in the mild category compared to flies fed on regular food. Feeding on Madhuyashti however, had a more dramatic effect than Guduchi, as maximum numbers of flies were observed in the severe category with acute necrotic patches, while only a few were observed to have moderate eye phenotype (Fig. 1B). The mild category was much less in Madhuyashti compared to Guduchi. These observations made it apparent that feeding larvae with either of the formulations increased the polyQ mediated neurodegeneration, with Madhuyashti proving to be more detrimental than Guduchi.

### Formulation feeding enhances rhabdomere disruption, aggregate formation, and axonal connections following expanded polyQ expression

**Fig. 2:**
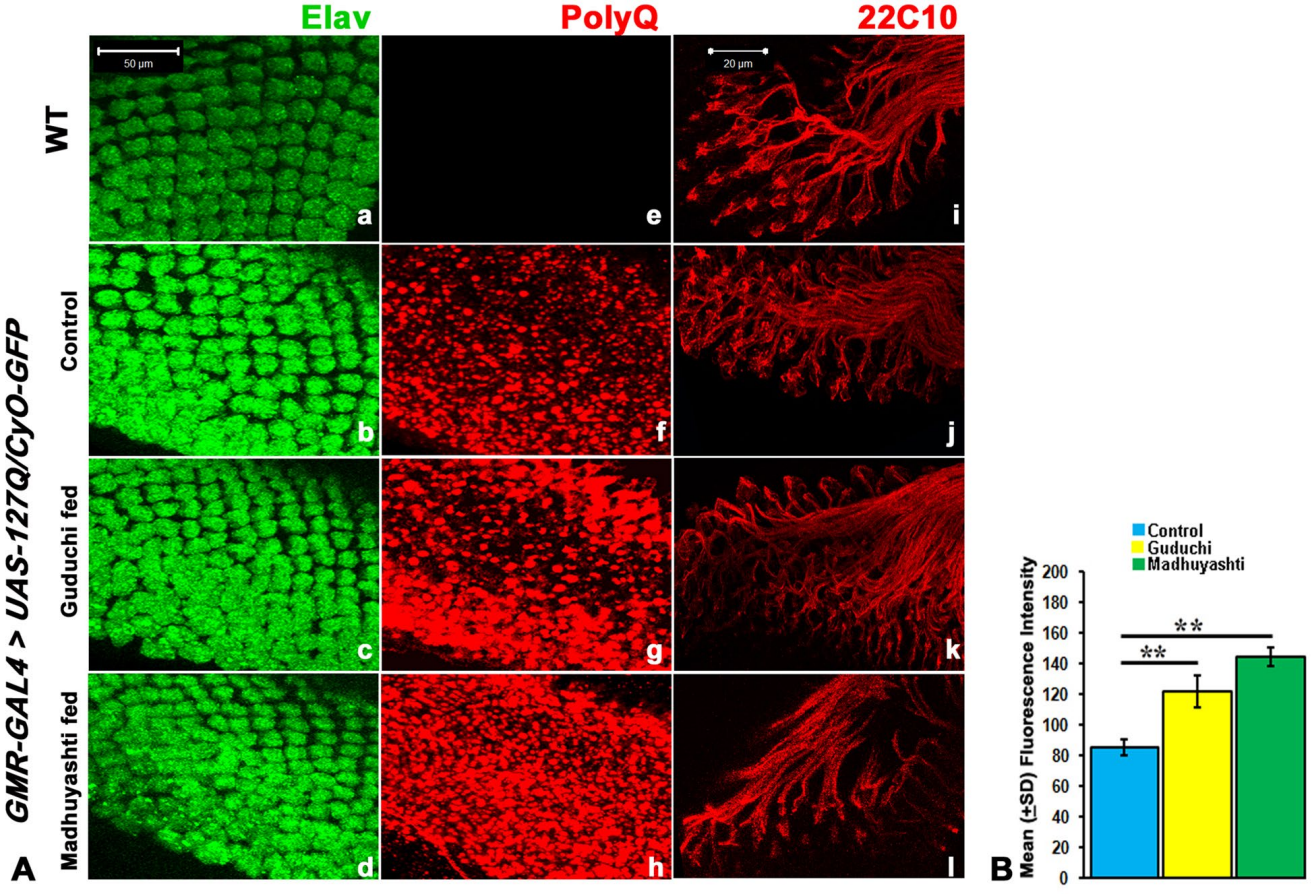
Formulation feeding disrupts rhabdomere arrangements, increases polyQ inclusion bodies and axonal connections **A** Confocal projection images show larval **e**ye imaginal discs immunostained with antibodies against anti-ELAV antibody (green) in *WT* (**a**) or *GMR-GAL4 > UAS-127Q/CyO-GFP* larvae fed on regular food (**b**) Guduchi (**c**) or Maduyashti (**d**) and polyQ (red) in *WT* (e) or *GMR-GAL4 > UAS-127Q/CyO-GFP* larvae fed on regular food (**f**) Guduchi (**g**) or Madhuyashti (**h**). Scale bar 50 μm, in section a is applicable to sections a to h. Immunostaining of eye imaginal discs with mab-22c10 antibody showed a regular arrangement of neuronal connections in *WT* (**i**), which was disrupted in *GMR-GAL4 UAS-127Q/CyO-GFP* larvae fed on regular food (**j**) and increase in disruption in Guduchi (**k**), or Madhuyashti supplemented food (**l**). Scale bar in section i, 20 μm is applicable to sections i to l. **B** Graph represents the mean (±SD) fluorescence intensities of IBs (Y-axis) in different feeding regimes, drawn through LSM 510 Meta. **represents *P* ≤ 0.001 using Student’s t test. The blue, yellow and green bars represent regular food, Guduchi and Madhuyashti respectively

Having observed enhanced degeneration in adult eyes, we looked at other markers to establish beyond doubt the detrimental effect of Ayurvedic formulations on Huntington’s phenotype (Fig. 2A). We first checked the ommatidial arrangement by neuronal marker, ELAV, which showed regular ommatidial arrangement in the eye imaginal discs of wild type (a), but not in *GMR-GAL4 > UAS-127Q/CyO-GFP* when fed on normal food (b). However, after feeding on Guduchi (c) and Madhuyashti (d), enhanced disruption in the rhabdomere arrangement was visible. We next checked the inclusion bodies, which are the hallmark of Huntington’s disorder, and the severity of degeneration is directly proportional to their size and number. As expected, aggregates were not present in the wild type (e) but were observed in *GAL4 > UAS-127Q/CyO-GFP* larvae fed on normal food (f). There was a marked increase in the number and size of the aggregates in the formulation fed larvae compared to control food. Among the formulation fed, Madhuyashti feeding (h) showed a substantially high accumulation of IBs compared to Guduchi (g), though both were considerably higher than regular food (f). This was further confirmed through the mean fluorescence intensity graph (Fig. 2B). Next, we checked the axonal connections of optical neurons, by immunostaining with mab22C10 antibody. In wild type larval imaginal discs regular arrangement of the axonal connection emerged from eyes discs and reached to the brain (i). It was clear that axonal projections were highly disarrayed in G*MR-GAL4 > UAS-127Q/CyO-GFP* larvae that were reared on normal food (j). The disruption was consequently more severely affected when the larvae were fed on Guduchi (k) or Madhuyashti (l) supplemented food, suggesting an increase in the disorganization of the axonal connections upon formulation feeding. All these observations correlated well with the enhanced neurodegeneration following formulation feeding.

### Formulation feeding impairs ubiquitin-Proteosome activity in *GMRGAL4 > UAS-127Q/CyO-GFP* expressing eye discs

**Fig. 3:**
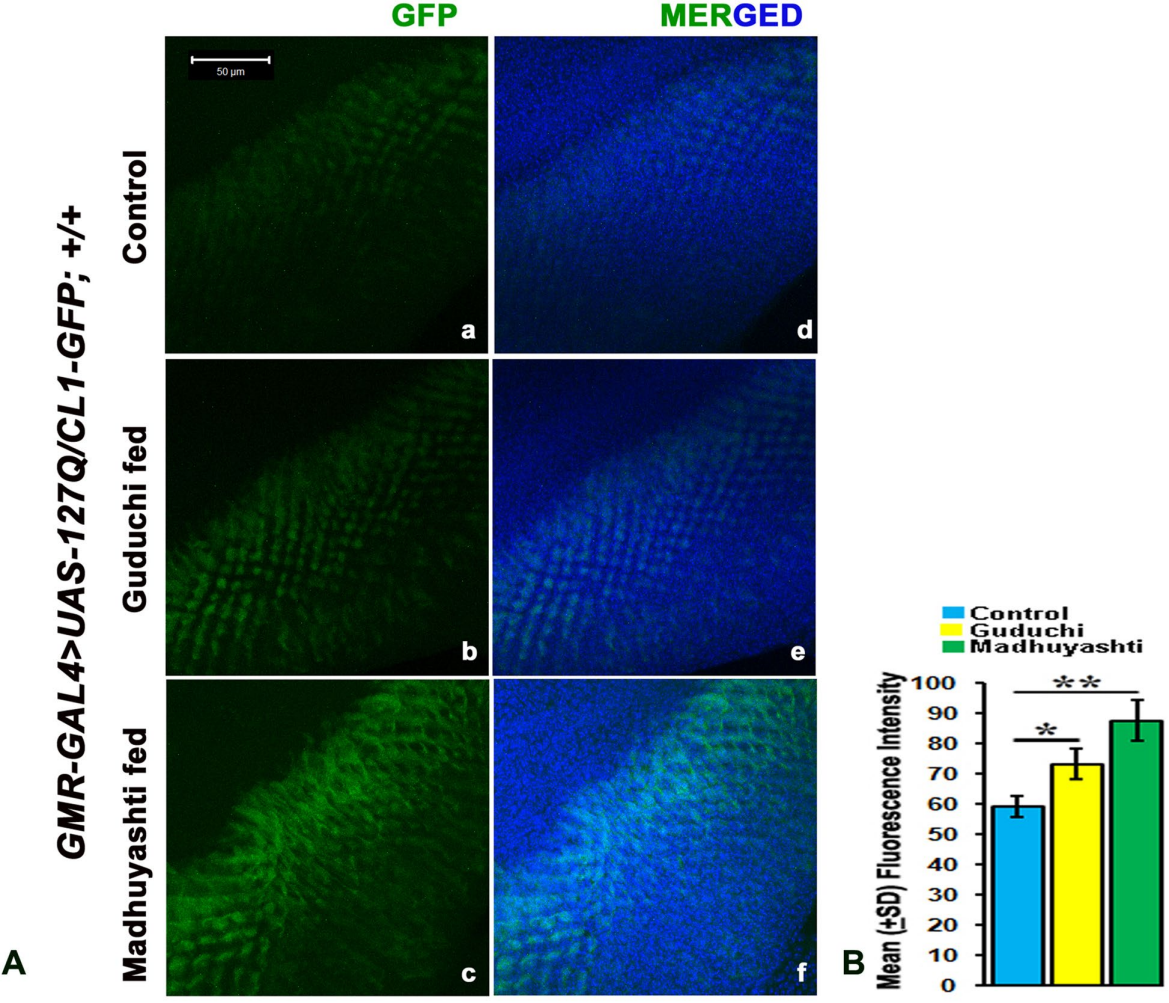
Formulation feeding degrades UPS activity in *-127Q* expressing eye discs. Confocal projection images showing larval eye imaginal discs expressing CL1-GFP when fed on regular food (**a**) Guduchi (**b**) or Madhuyashti (**c**). Nuclei counterstained with DAPI (blue) (**d**, **e**, **f**). Scale bar in a, 50 μm is applicable to all images from **a**-**f**. **B** Graph represents the mean (±SD) fluorescence intensities of CL1-GFP (Y-axis) in different feeding regimes, drawn through LSM 510 Meta. The blue, yellow and green bars represent control, Guduchi and Madhuyashti respectively. ** represents *P* ≤ 0.001 and * represents *P* ≤ 0.05 using Student’s t test

Ubiquitin–proteasome pathway (UPS) is essential for the rapid degradation of many critical regulatory proteins, its disruption is fatal for the cells. To evaluate UPS function under various feeding regimes, transgenic flies expressing a fluorescent reporter of UPS function *UAS-CL1-GFP,* a fusion protein created by introducing a degradation signal to otherwise stable green fluorescent protein (GFP) was used [25]. This protein is rapidly degraded by the UPS, and its steady-state levels reflect the functional status of this pathway. *GMR-GAL4 > UAS-127Q/UAS-CL1-GFP* larvae (Fig. 3A) when fed on regular food (a) showed GFP signal, reflecting impaired UPS. This signal was intensified when larvae were fed on Guduchi (b) and increased further on Madhuyashti (c), showing enhanced degradation of UPS machinery. This was also validated through the mean fluorescence intensity graph (Fig. 3B). Un-driven third instar larvae does not show GFP signals in any of the feeding regimes (Supplementary Fig. 1).

### Elevation of the immune response following Guduchi or Madhuyashti feeding aggravates polyQ mediated degeneration

Guduchi and Madhuyashti have been shown to activate immune response pathways [37], we analyzed the AMP production as well as immune pathway genes in polyQ expressing larvae following formulation feeding. RT-PCR analysis showed increased expression of all AMPs, *AttacinA*, *CecropinA*, *Defensin*, *Drosomycin*, *Diptericin*, *Drosocin*, and *Metchnikowin*, upon formulations feeding suggesting that increased AMPs do have a bearing on the enhanced degeneration. (Table 1).

**Table 1:** Mean fold change in the expression of AMPs upon formulation feeding

AMPs	Mean -∆∆CT values (**+**SD; ***N*** = 3 biological replicates in each) of expression of various AMPs with respect to ***GMR-GAL4 > UAS-127Q/CyO-GFP*** larvae fed on regular food (> 2 ∆∆CT value is considered significant i.e., ***p*** < 0.05 and is marked with *)	
	Guduchi	Madhuyashti
**AttacinA**	3.42 + 0.40*	4.23 + 0.03 *
**CecropinA**	2.34 + 0.05*	2.78 + 0.05*
**Defensin**	2.03 + 0.02*	2.56 + 0.3*
**Drosomycin**	3.89 + 0.24*	2.68 + 0.03*
**Diptericin**	4.28 + 0.45*	3.86 + 0.56 *
**Drosocin**	2.67 + 0.78*	3.23 + 0.67 *
**Metchnikowin**	2.76 + 0.65*	2.83 + 0.54 *

When the expression of other genes in the Toll-pathway were analyzed, contrary to the expectation, except Spatzle*,* which was enhanced, no change in transcript levels of either *MyD88*, *Tube*, *Pelle*, *Cactus* or *Dorsal* were observed (Table 2) upon formulation feeding, as compared to those reared on regular food in *GMR-GAL4 > UAS-127Q/CyO-GFP,* suggesting that the expression of Attacin, Drosocin, and Drosomycin are not governed by the canonical signaling pathways.

**Table 2:** Mean fold change in the expression of genes involved in Toll-pathway upon formulation feeding

Genes	Mean -∆∆CT (**+**SD; ***N*** = 3 biological replicates in each) of expression of various upstream genes of Toll-pathway in ***GMR-GAL4 > UAS-127Q/CyO-GFP*** with respect to larvae fed on regular food (> 2 ∆∆CT value is considered significant i.e., ***p*** < 0.05 and is marked with *)	
	Guduchi	Madhuyashti
Dorsal	1.43 + 0.07	1.33 + 0.1
Spatzle	2.45 + 0.4*	3.68 + 0.4*
Myd88	0.84 + 0.03	0.73 + 0.13
Cactus	0.34 + 0.11	0.54 + 0.09
Tube	0.59 + 0.34	0.63 + 0.16
Pelle	0.83 + 0.45	0.67 + 0.22

Similar to the Toll pathway, in the IMD pathway except for *Imd* and *Tak1*, none of the other genes of the IMD pathway were activated upon formulation feeding (Table 3). The most puzzling observation was the lack of increased expression of transcription factors Relish and Cactus,

**Table 3:** Mean fold change in the expression of genes involved in IMD-pathway upon formulation feeding

Genes	Mean -∆∆CT (**+**SD; ***N*** = 3 biological replicates in each) of expression of genes of IMD-pathway in ***GMR-GAL4 > UAS-127Q/CyO-GFP*** with respect to larvae fed on regular food (> 2 ∆∆CT value is considered significant i.e., ***p*** < 0.05 and is marked with *)	
	Guduchi	Madhuyashti
Tab2	0.96 + 0.06	1.2 + 0.26
Imd	3.48 + 0.17*	3.87 + 0.4*
Fadd	1.67 + 0.04	1.35 + 0.07
Dredd	0.89 + 0.33	0.98 + 0.52
Tak1	4.67 + 0.33*	4.38 + 0.23*
Relish	1.36 + 0.56	1.23 + 0.32

which indicated that AMPs were activated independently of NF-κB transcription factors. The expression of Relish was further confirmed by immunostaining (Fig. 4A), which was increased in *GMR-GAL4 > UAS-127Q/CyO-GFP* larval imaginal discs (c) as compared to *WT* (a). However, feeding with Guduchi (e) or Madhuyashti (g) did not further elevate the levels of Relish, and it remained the same as *GMR-GAL4 > UAS-127Q/CyO-GFP,* even though there was enhanced expression of AMPs. Nuclei were counterstained with DAPI, as can be seen in merged images (b, d, f, h). The expression of Relish was also quantified and no significant difference between the two formulations was observed and they were equivalent to what was observed in regular food (Fig. 4B).

**Fig. 4:**
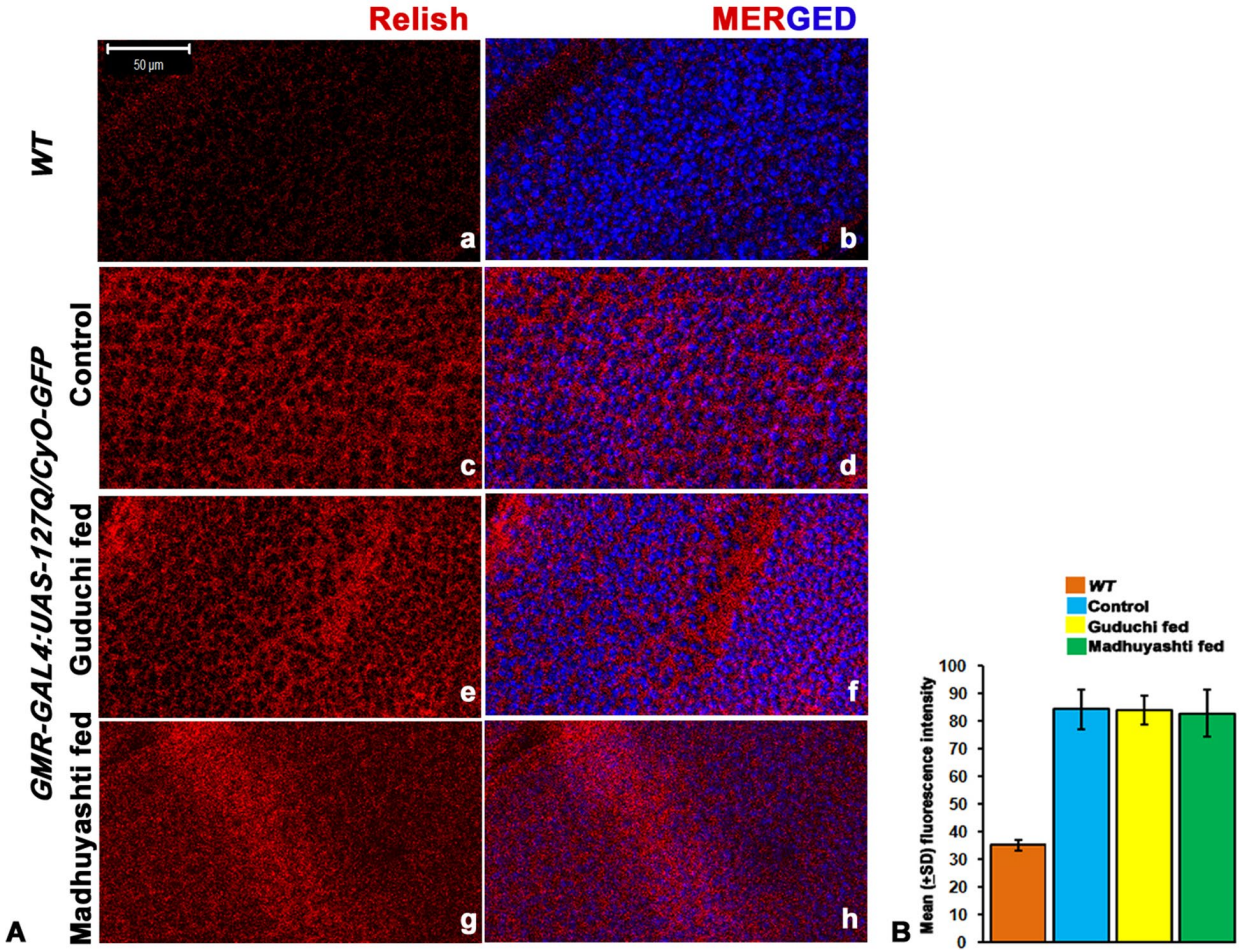
Formulation feeding does not alter Relish expression levels in *GMR* driven *-127Q* expressing eye discs. Confocal projection images showing larval eye imaginal discs stained with anti-Relish in *WT* eye imaginal disc (**a**) or *GMR-GAL4 > UAS-127Q* larval discs when fed on regular food (**c**) Guduchi (**e**) or Madhuyashti (**g**). Nuclei counterstained with DAPI (blue) (**b**, **d**, **f**, **h**). Scale bar in section A is 50 μm and is applicable to all sections from **a-h**. **B** Graph represents the mean (±SD) fluorescence intensities of Relish (Y-axis) in different feeding regimes, drawn through LSM 510 Meta. The blue, yellow, and green bars represent regular food, Guduchi and Madhuyashti respectively

### Down-regulation of AMPs but not relish rescued polyQ mediated neurodegeneration in formulation fed condition

**Fig. 5:**
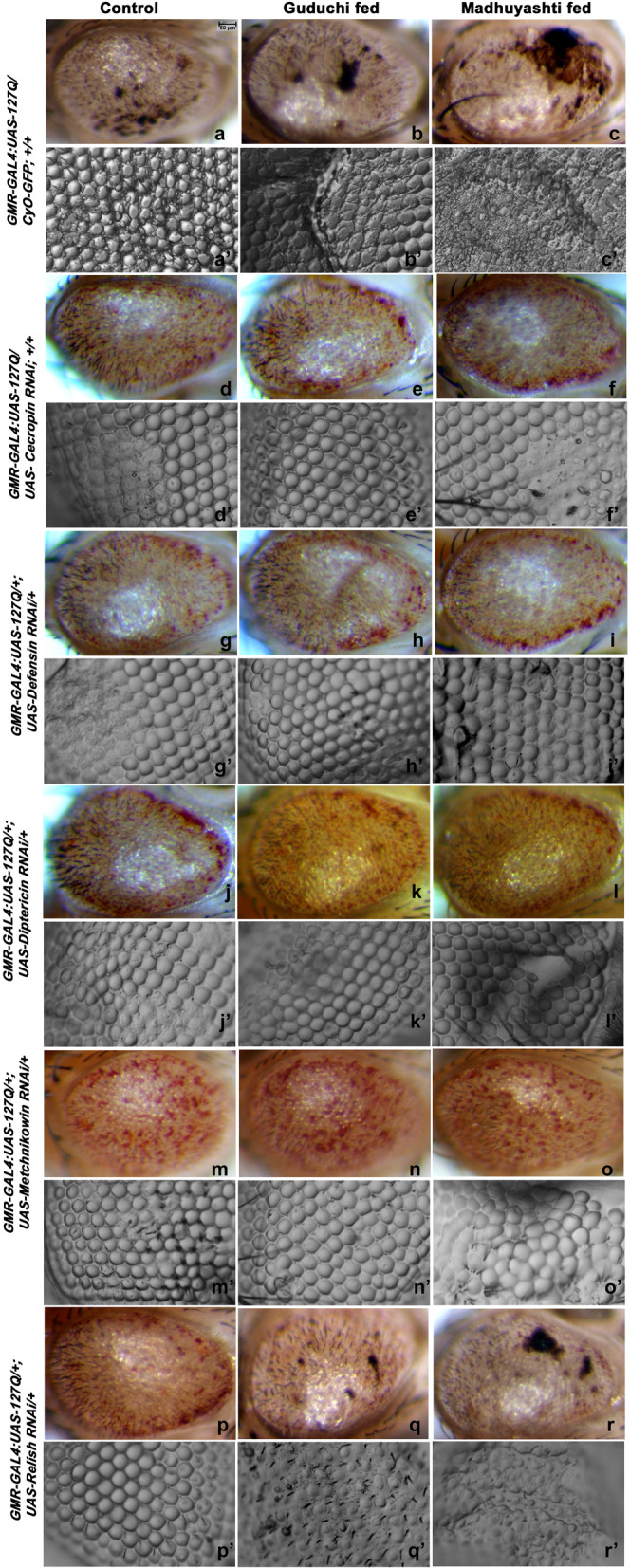
Down regulation of AMPs rescued polyQ mediated neurodegeneration. Panel represents eye image along with respective eye imprints (below each image) (a’-r’) in *GMR-GAL4:UAS-127Q/CyO-GFP* **(a-c)***, GMR-GAL4:UAS-127Q/UAS-Cecropin RNAi;+/+*(**d**-**f**), *GMR-GAL4:UAS-127Q/+;UAS-Defensin-RNAi/+*(**g-i**)*, GMR-GAL4:UAS-127Q/+;UAS-Diptericin RNAi/+* (**j-l**), *GMR-GAL4:UAS-127Q/+;UAS-Metchnikowin RNAi/+*(**m-o**), *GMR-GAL4:UAS-127Q/+;UAS-Relish RNAi/+* (**p-r**) under various feeding regimes. Scale bar in section a 20 μm in a is applicable to images **a-r** and **a’-r’**

As depletion of AMPs can attenuate polyQ phenotype, we then down-regulated AMPs, one at a time and observed the effect on degeneration in different feeding regimes (Fig. 5). Down-regulation of four AMPs, CecropinA (d-f), Defensin (g-i), Diptericin (j-l), and Metchnikowin (m-o) was concomitant with rescue in polyQ mediated neurodegenerative phenotype as compared to *GMR-GAL4 > UAS-127Q* (a-c). It was also evident that the rescue was to the same extent in all three feeding regimes i.e., regular food (d, g, j, m), Guduchi (e, h, k, n), or Madhuyashti (f, i, l, o), which was further confirmed through nail polish imprints (a’-o’). However, down-regulation of Relish was only effective in rescuing flies fed on regular food (p), and had less effect on formulation fed flies (q, r), where 42% of flies showed mild and 35% of flies showed moderate phenotype when fed on food supplemented with Guduchi, while 40% of flies showed mild and 40% of flies showed moderate phenotype when fed on Madhuyashti, although no severe phenotype was observed. These results confirmed that the AMPs were not unregulated by the canonical pathway.

### JNK pathway gets activated following formulation feeding

JNKs family of protein kinases activates the IMD pathway in *Drosophila* and leads to the transient expression of AMPs, and once Relish is activated, this transient response ceases [28]. TAK1, a MAPKKK, in the IMD pathway is known to activate Hemipterous, a MAPKK, which in its down cascade leads to activation of the basket (MAPK), *Drosophila* homologue of JNK [4, 8]. Thus, transcript levels of these genes were checked and they showed an increase in formulation fed food compared to regular food. Among the two formulations, the increase in expression was more in Madhuyashti as compared to Guduchi (Table 4).

**Table 4:** Mean fold change in the expression of *Hemipterous* and *basket* genes upon formulation feeding

Genes	Mean -∆∆CT (**+**SD; ***N*** = 3 biological replicates in each) of expression of ***Tak1*** and ***basket*** genes in ***GMR-GAL4 > UAS-127Q/CyO-GFP*** with respect to larvae fed on regular food (> 2 ∆∆CT value is considered significant i.e., ***p*** < 0.05 and is marked with *)	
	Guduchi	Madhuyashti
Hemipterous	2.63 + 0.03*	3.97 + 0.04*
Basket	3.38 + 0.06*	4.09 + 0.35*

**Fig. 6:**
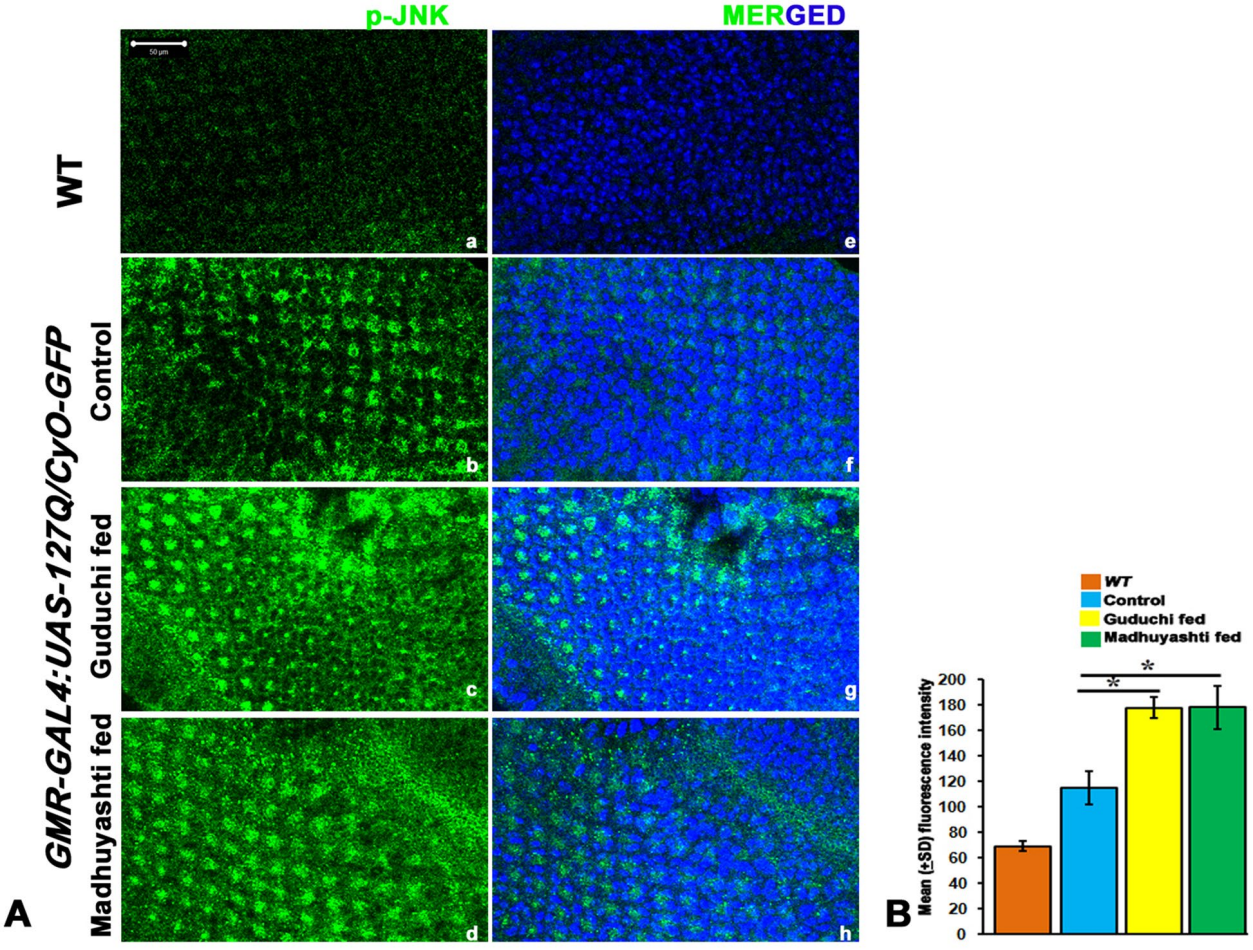
Formulation feeding elevated JNK expression levels *-127Q* expressing eye discs. Confocal projection images showing larval eye imaginal discs stained with anti-p-JNK in *WT* eye imaginal disc (**a**) or *GMR-GAL4 > UAS-127Q/CyO-GFP* larval discs when fed on regular food (**b**) Guduchi (**c**) or Madhuyashti (**d**). Nuclei counterstained with DAPI (blue) (**e**, **f**, **g**, **h**). Scale bar in section A, 50 μm is applicable to all sections from **a**-**h**. **B** Graph represents the mean (±SD) fluorescence intensities of JNK (Y-axis) in different feeding regimes, drawn through LSM 510 Meta. The blue, yellow and green bars represent control, Guduchi and Madhuyashti respectively. The blue, yellow, and green bars represent control, Guduchi and Madhuyashti respectively. * represents *P* ≤ 0.05 using Student’s t test

**Fig. 7:**
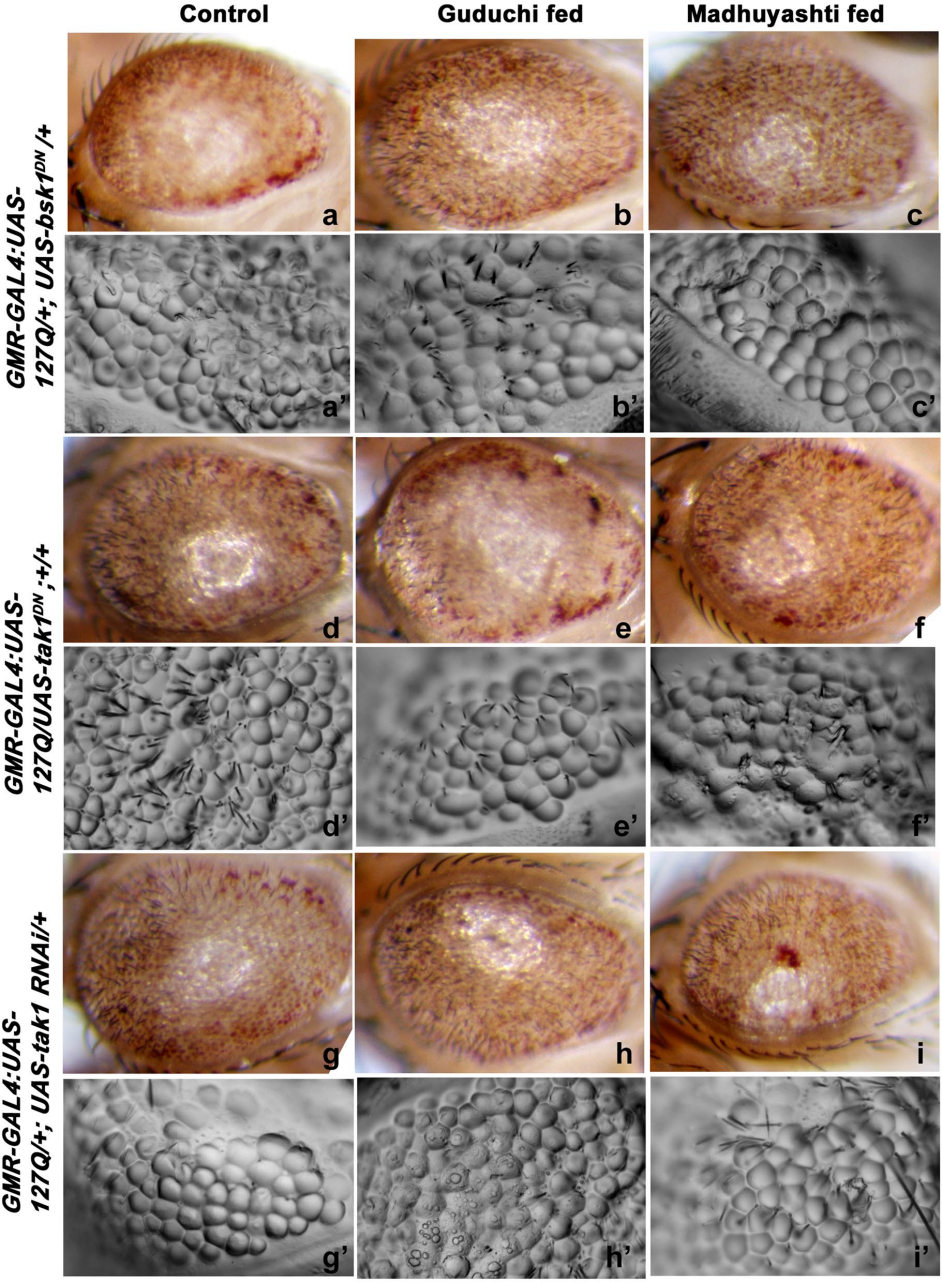
Down regulation of Tak1 or Basket rescued polyQ mediated neurodegeneration. Panel represents eye image (**a**-**i**) along with respective eye imprints (below each image) (**a’-i’**) in *GMR-GAL4:UAS-127Q/+;UAS-bsk*^*DN*^*/+*, (**a**-**c**)*, GMR-GAL4:UAS-127Q/+;UAS-Tak1 RNAi/+*(**d**-**f**), *GMR-GAL4:UAS-127Q/UAS-Tak1*^*DN*^; *+/+* (**g**-**i**) under various feeding regimes. For each genotype under all three feeding regimes > 150 flies were observed and the experiment was done in three independent replicates. Scale bar in section a 20 μm in a is applicable to images **a-i** and **a’**-**i’**

Since transcription of JNK was enhanced, we further confirmed it by looking at the protein levels by immunostaining (Fig. 6A). Expression of JNK was elevated in polyQ conditions fed on regular food (b) as compared to WT (a). Following the same pattern as observed for transcripts, feeding with Guduchi (c) or Madhuyashti (d) further elevated protein levels of JNK, which was further confirmed through the mean fluorescence intensity graph drawn through LSM10 Meta (Fig. 6B). Nuclei were counterstained with DAPI as represented in the merged section (e, f, g, h). Since components of the JNK signaling pathway, *Tak1* and *basket* were elevated, we next validated their contribution genetically by observing the consequences of reducing their levels by RNAi under three feeding regimes (Fig. 7). The rescue was accomplished when *basket* dominant-negative mutant was used to inhibit Basket function in *GMR-GAL4 > UAS-127Q/+; UAS-bsk*^*DN*^*/+*, under all the three feeding regimes (a, b, c). Similarly, we also observed a significant rescue when *Tak1* expression was down-regulated in *GMR-GAL4:UAS-127Q/+;UAS-Tak1 RNAi/+* or *GMR-GAL4:UAS-127Q/UAS-Tak1*^*DN*^; *+/+*, and the rescue was irrespective of their feeding conditions i.e. regular (d, g), Guduchi (e, h) or Madhuyashti (f, i). The above results were further confirmed through nail polish imprints which showed improved ommatidial arrangements when compared to those in *GMR-GAL4 > UAS-127Q/CyO-GFP* (Fig. 7 a’, b’, c’) under three feeding regimes. Taken together these findings suggest the possibility that Madhuyashti and Guduchi execute their actions via the JNK pathway.

Since polyQ mediated pathogenicity is associated with the accumulation of inclusion bodies, we checked their expression in AMPs, Basket, Tak1, and Relish down-regulated background by staining with the anti-HA antibody. Under conditions where rescue was observed, reduced expression of IBs was noticed, except for Relish, which did not show the same reductions as rest (data not shown).

### Formulation feeding elevated the caspases levels in the eye discs of *GMR-GAL4 > USA-127Q/CyO-GFP* larvae

**Fig. 8:**
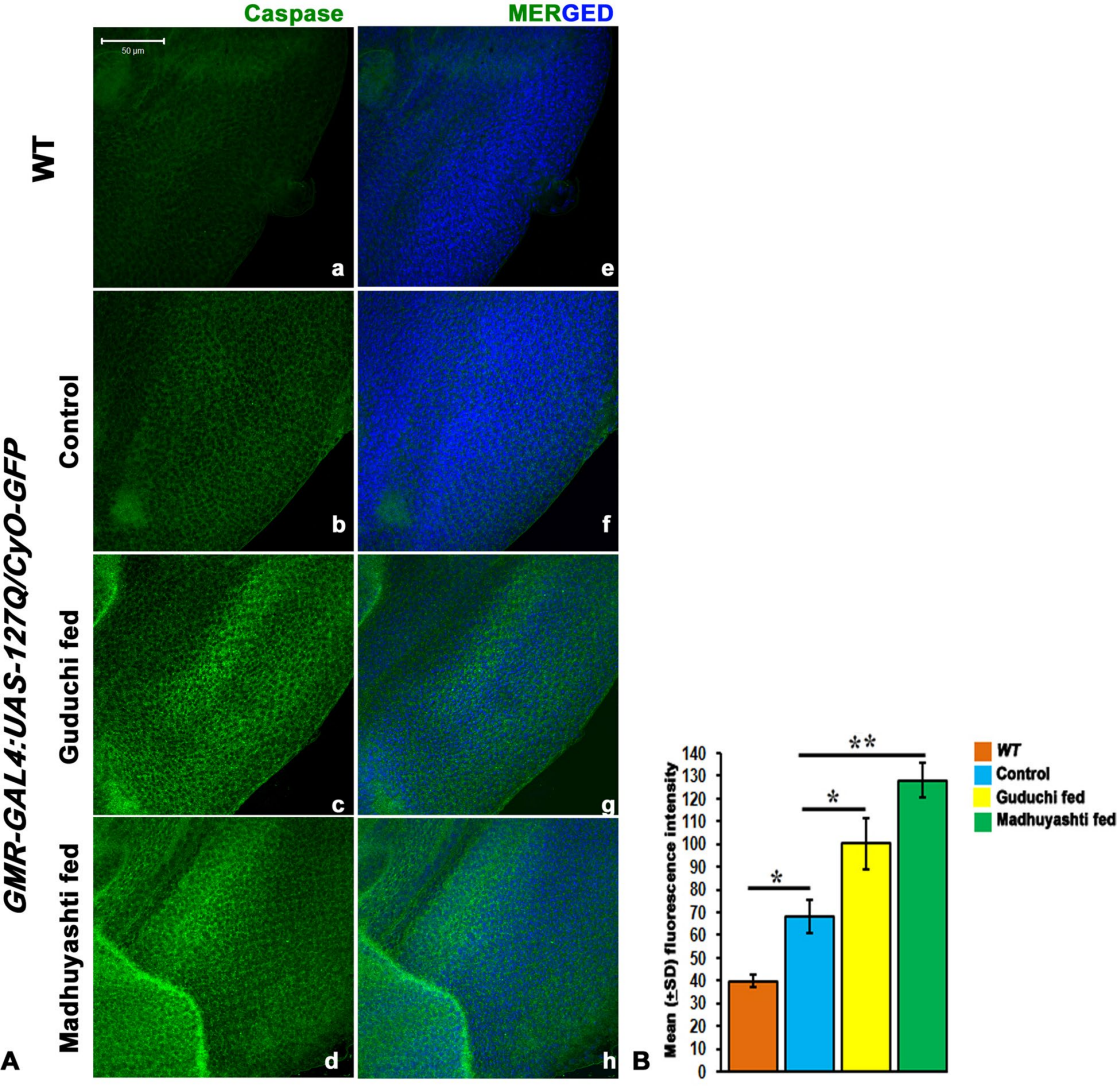
Formulation feeding elevated Caspase expression in GMR-GAL4driven *127Q* expressing eye discs. Confocal projection images showing larval eye imaginal discs stained with anti-Caspase3 in *WT* eye imaginal disc (**a**) or *GMR-GAL4 > UAS-127Q/CyO-GFP* larval discs when fed on regular food (**b**) Guduchi (**c**) or Madhuyashti (**d**). Nuclei counterstained with DAPI (blue) (**e**, **f**, **g**, **h**). Scale bar in section A, 50 μm is applicable to all sections from **a**-**h**. **B** Graph represents the mean (±SD) fluorescence intensities of Caspase3 (Y-axis) in different feeding regimes, drawn through LSM 510 Meta. The blue, yellow and green bars represent control, Guduchi and Madhuyashti respectively. ** represents *P* ≤ 0.001 and * represents *P* ≤ 0.05 using Student’s t test

In addition to immunity, JNK enhanced expression also causes apoptosis [20, 23, 27]. Since JNK expression was elevated, active caspase expression was checked using an anti-caspase antibody (Fig. 8A). It was observed that the expression of total Caspase3 was elevated in polyQ conditions fed on regular food (b) as compared to WT (a). Feeding Guduchi (c) or Madhuyashti (d) further elevated Caspase3 levels which was further confirmed through the mean fluorescence intensity graph drawn through LSM10 Meta (Fig. 8B). Nuclei were counterstained with DAPI as represented in the merged section (e, f, g, h).

Since there was an increase in the total activated Caspase levels, which is a clear indicator of apoptosis, we counterchecked it with the number of proliferating cells. The *GMR-GAL4 > UAS-127Q/CyO-GFP* larval discs were immunostained with Phosphohistone3 (PH3) antibody, a mitotic cell proliferation marker and by counting the number of cells. There was a significant decrease in the number of proliferating cells (data not shown).

## Discussion

Ayurveda refers to the intake of certain plant products, believed to have a predominantly beneficial effect on humans, however, several studies indicated undesired results of Ayurvedic formulations [32]. Studies conducted by [18], suggested side effects of several plant products including *Tinosopra cordifolia*, *Piper longum*, *Withania somnifera*, *Ocimum sanctum,* etc. The unexpected effects could be because the understanding of the molecular mechanisms underlying the effects of different traditional medicines is inadequate, which may sometimes lead to an unanticipated result, thus putting a question mark about the intake of these medicines for all ailments.

In the present study, we state that intake of Guduchi and Madhuyashti at the dosage used under conditions of Huntington’s disease, does not lead to amelioration of the disease, instead causes more harm, an observation which is contradictory with the known notions about the goodness of these formulations. Among the two formulations, Madhuyashti being more detrimental, suggests that the formulations are governed by complex principles and mechanisms and it needs to be understood that Ayurvedic formulation and their extracts act differently under different conditions. Even though Ayurvedic formulations are based on natural herbal materials, their safety depends on their method of administration, taking into account individuals’ needs and their specific disease conditions [18].

In our study, we observed a positive correlation between the expressions of JNK and enhanced humoral innate immune response when exposed to Ayurvedic formulations. The existence of crosstalk between NF-κB and JNK is evolutionarily conserved [27], suggesting a fine regulation of immune activation upon pathogenic challenge. In *Drosophila*, the duration of JNK induction in response to LPS is directly controlled by Relish which attenuates JNK cascade soon after its activation [27, 28], thus preventing erroneously triggering of apoptosis following JNK activation. *Drosophila* TNF-α homolog, Eiger, depends on JNK – rather than caspase-8 homolog, Dredd – to induce death [16, 24]. Thus, the role of JNK in apoptosis signaling appears to be a remnant of a primordial death mechanism engaged by TNF-α, which only later in evolution began to exploit the FADD/caspase-dependent pathway, yet maintaining its connection to NF-κB, thus establishing the fact that Relish is essentially required for attenuation of JNK. Apart from this, JNK signaling has been recognized for its diverse roles in cellular events such as cell migration, dorsal closure, regeneration, [22] tumorigenesis, and neurodegenerative disorders [10, 42], and NF-κB/Relish participates in a wide range of biological processes, including immunity, lymphopoiesis, epidermal homeostasis, and development of hair follicle and central nervous system [2, 26]. We provide evidence that these formulations are disrupting this crosstalk resulting in sustained expression of AMPs by the JNK pathway and not through NF-κB transcription factors.

Innate immune response in *Drosophila* during the pathogenic challenge is initially transiently expressed by the activation of JNK (basket) by Tak1. Once the Basket is phosphorylated, it activates AMP genes. One possible reason for this transient response in invertebrates could be because activation by phosphorylation of JNK is a rapid process and it acts faster than activation of Relish, which is first cleaved and then translocated into the nucleus for a sustained response [15]. As soon as Relish activation is consistent, the stability of TAK1 is greatly decreased, thus inhibiting the continuous activation of JNK, suggesting that certain downstream targets of Relish facilitate the destruction of TAK1 and switch off the JNK cascade [11, 28].

**Fig. 9:**
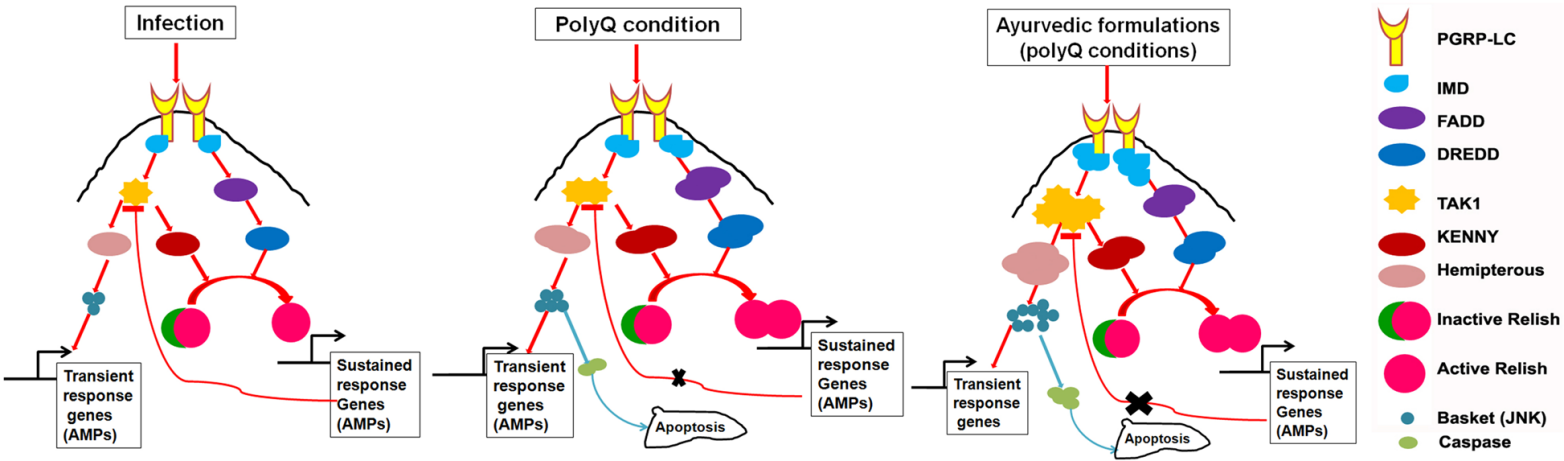
Schematic representation of molecular effects in normal and polyQ conditions, upon feeding on Guduchi and Madhuyashti

In polyQ conditions, there is an increase in immune response [12]. When transcripts of all these genes were compared between polyQ conditions under various feeding regimes, we can see that there is an increase in the levels of *IMD*, *Tak1, Hemipterous,* and *basket*, whereas all other genes are comparable to those of polyQ unfed condition. Therefore, it could be possible that Relish fails to suppress Tak1 and along with the sustained response, there is a continuously active response, which finally leads to elevation of immune response causing exaggeration of neurodegeneration (Fig. 9). Another observation that correlates with the study is the elevated expression of Caspase, leading to apoptosis and decreased number of proliferating cells in fed conditions. Thus, there could be a possibility that the number of actively diving cells is less and those undergoing apoptosis are more, which causes more degeneration in the formulation fed polyQ larvae/flies.

Relish expression levels remain unaltered, it fails to suppress Tak1 (negative feedback), thus the functional relevance of formulation feeding can be important for understating their use in therapeutic applications. These findings reveal an unanticipated cross-talk between *Drosophila* immune response and neurodegeneration upon formulation feedinghold promise for mechanistic insight into the mammalian counterpart of this regulatory interaction.

In our study, some results are not in agreement with the previous studies [17, 35] in mouse/cell lines. This could be possible due to several reasons involved such as dose administered, active components, region for plant collection, method of preparation and method of administration.

Targeting JNK signaling constitutes an obvious opportunity for therapeutic intervention. However, MAPK inhibitors can display toxic effects, as it is essential for various cellular processes. Consequently, dual-specificity MKKs (MAPK kinases) may represent more attractive targets. In particular, evidence that blocking JNK activation by removing MKK4 offers an effective therapy to treat pathological conditions has started to emerge [10]. This also indicates cautious use of the formulations and there need to be more studies to understand better the implications of these Ayurvedic formulations.

## Conclusion

The present study provides ample evidence that the two formulations Guduchi and Madhuyashti exaggerate polyQ mediated neurodegeneration, which is mediated through elevated immune response via JNK signaling, instead of canonical Relish mediated immune response. This study indicates that additional in-depth unbiased scientific research needs to be conducted for a better understanding of molecular mechanisms underlying the therapeutic applications of Ayurvedic formulations.

## Supplementary Information

**Additional file 1: Supplementary Fig. 1.** *GMR-GAL4* driven *UAS-CL1-GFP* doesn’t show GFP signal under either of the feeding regimes indicating that UPS is active functional.

## Data Availability

The datasets used and/or analysed during the current study are available from the corresponding author on reasonable request.

## Abbreviations

IBs: Inclusion bodies
IQ: Intelligence Quotient
HD: Huntington’s Disease
AMP: Anti-Microbial Peptide
IMD: Immune Deficiency
JNK: Jun N-terminal Kinase
