# Supplementary figures and images for: Ayurvedic formulations Guduchi and Madhuyashti triggers JNK signaling mediated immune response and adversely affects Huntington phenotype
Source: BMC Complement Med Ther. 2022 Oct 12;22:265. doi: 10.1186/s12906-022-03724-9 (PMC9555103; doi:10.1186/s12906-022-03724-9)

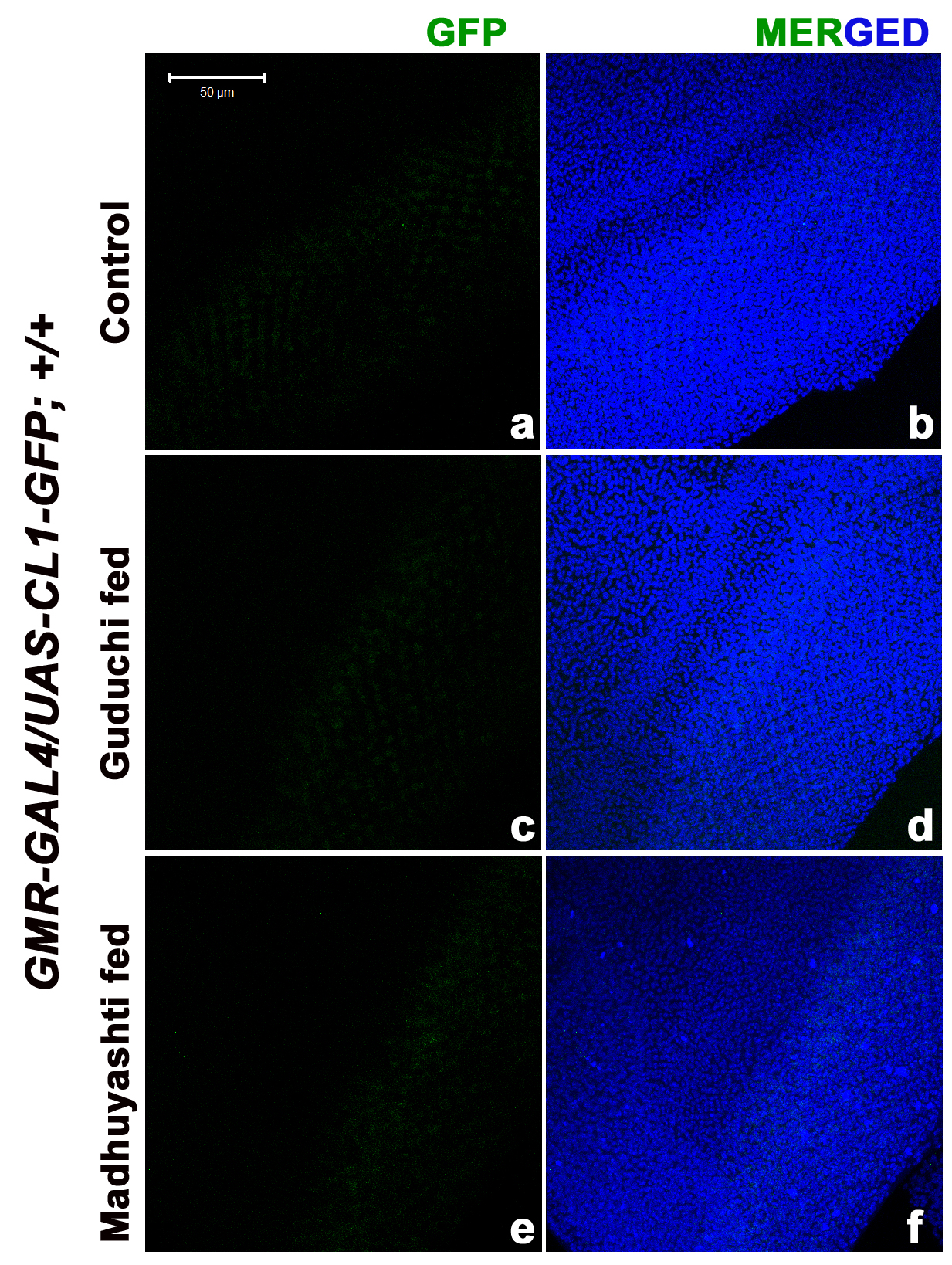

Supplement: Supplementary file 1 — Additional file 1: Supplementary Fig. 1. GMR-GAL4 driven UAS-CL1-GFP doesn’t show GFP signal under either of the feeding regimes indicating that UPS is active functional. [file 12906_2022_3724_MOESM1_ESM.jpg]
